# 

**Published:** 1904-09

**Authors:** 


### CONTENTS.

| | PAGE |
|---|---|
| **Obituary—Edward Crossman, M.D. Durh.** | 193 |
| **Lac Vinum Infantum: A Review of the Work of Infant Milk Depots.** By J. M. FORTESCUE-BRICKDALE, M.A., M.D. Oxon. | 196 |
| **Some Observations on Tuberculous Disease of the Hip-Joint in Childhood and Youth.** By CHARLES A. MORTON, F.R.C.S. | 207 |
| **On the Throat as the Source of Systemic Infection in Acute Rheumatism.** By P. WATSON WILLIAMS, M.D. Lond. | 215 |
| **The Indications for Operation in Myo-Fibroma of the Uterus.** By JAMES SWAIN, M.S., M.D. Lond., F.R.C.S. | 220 |
| **Ethyl-Chloride: A few Practical Remarks.** By A. L. FLEMMING | 228 |
| **The Induction of General Anæsthesia by Intra-Spinal Injections of Cocaine.** By ALFRED S. GUBB, M.D. Paris, M.R.C.S., &c. | 231 |
| **On Surgical Analgesia by Spinal Cocainisation.** By WILLIAM JONES GREER, F.R.C.S.I., D.P.H.I. | 235 |
| **The Relationship of Chorea and Rheumatism.** By JOS. J. S. LUCAS, M.B., B.A. Lond. | 239 |
| **Hæmatemesis Associated with Small White Kidneys.** By THEODORE FISHER, M.D., M.R.C.P. | 243 |
| **Notes on a Case of Infantilism.** By E. CECIL WILLIAMS, B.A., M.B. Cantab. | 247 |
| **Progress of the Medical Sciences:** | |
| **Medicine.** G. PARKER, M.D. Cantab. | 249 |
| **Surgery.** T. CARWARDINE, M.S. Lond., F.R.C.S. | 253 |
| **Reviews of Books** | 257 |
| **Editorial Notes** | 268 |
| **Notes on Preparations for the Sick** | 276 |
| **The Library of the Bristol Medico-Chirurgical Society** | 283 |
| **Obituary—H. A. Benham, M.D. Aberd.** | 286 |
| **Local Medical Notes** | 288 |

